# Application of the human estrogen receptor within a synthetic transcription factor in *Trichoderma reesei*

**DOI:** 10.1186/s40694-020-00102-3

**Published:** 2020-08-03

**Authors:** Christian Derntl, Robert Mach, Astrid Mach-Aigner

**Affiliations:** Institute of Chemical, Environmental and Bioscience Engineering, TU Wien, Gumpendorfer Strasse 1a, 1060 Vienna, Austria

**Keywords:** *Trichoderma reesei*, Synthetic biology, Xyr1, Ypr1, Transcription factor, Estradiol, Gene expression, Human estrogen receptor α

## Abstract

**Background:**

Synthetic gene expression systems offer a possibility for controllable and targeted induction of the expression of genes of interest, which is a fundamental technique necessary for basic research and industrial applications. The human estrogen receptor α contains a ligand binding domain that enforces dimerization and nuclear import upon binding of the inducer 17β-estradiol. In this study, we tested the potential of this ligand binding domain to be used in filamentous fungi as an auto-regulatory domain in a synthetic transcription factor.

**Results:**

We constructed the synthetic transcription factor SynX by fusing the DNA-binding domain of Xyr1 (Xylanase Regulator 1), the transactivation domain of Ypr1 (Yellow Pigment Regulator 1), and the ligand binding domain of the human estrogen receptor α. SynX is able to strongly induce the gene expression of xylanases and an aldose reductase by addition of 17β-estradiol, but SynX does not induce gene expression of cellulases. Importantly, the induction of xylanase activities is mostly carbon source independent and can be fine-tuned by controlling the concentration of 17β-estradiol.

**Conclusion:**

The ability of SynX to induce gene expression of xylanase encoding genes by addition of 17β-estradiol demonstrates that the ligand binding domain of the human estrogen receptor α works in filamentous fungi, and that it can be combined with a transactivation domain other than the commonly used transactivation domain of herpes simplex virion protein VP16.

## Background

Controlled induction of gene expression is a necessity for basic research and industrial applications. An ideal expression system has no basal expression and can be induced by a stimulus that does not interfere with the native metabolism or regulatory network. Moreover, the expression should be tunable and reach a high expression level. Such a system enables the expression of genes of interest at a desired time point to desired intensities, allowing for example the expression of toxic or harmful proteins or the synthesis of toxic substances.

For filamentous fungi several such synthetic expression systems were developed previously. In 2005, the human estrogen receptor α (hERα) was used successfully in *A. niger* and *A. nidulans* and could drive gene expression from a synthetic promoter upon induction with estrogenic substances [1]. The hERα is a nuclear receptor reacting to a group of small, hydrophilic substances, including the hormone 17β-estradiol. The hERα consists of 5 structural domains (A/B, C, D, E, and F). The A/B domain supports the transactivation activity of domain E, Domain C is the DNA-binding domain (DBD), domain D is a linker containing a nuclear localization signal, domain E is the ligand binding domain (LBD), and domain F appears to modulate the transcriptional activity, co-activator interactions, dimerization, and stability of the receptor [2–12]. Upon binding of an inducer to the LBD, hERα undergoes a conformational change, which results in dimer stabilization, nuclear import, and exposure of surfaces for interactions with the basic transcription machinery (the LBD is also responsible for transactivation) [6, 7, 10, 13, 14]. Refer to the review by Yaşar et al. [15] for a summary about the current knowledge on the hERα.

Also in 2005, the Tet-on/Tet-off system, which had previously been successfully applied in several eukaryotic species [16–21], was demonstrated to work in *A. fumigatus* [22]. This system is based on the bacterial TetR repressor that regulates the tetracycline resistance operon in *Escherichia coli* by binding to the *tetO* sequence upon the presence of tetracycline or doxycycline. For applications in eukaryotes, TetR was fused to the transactivation domain (TAD) of herpes simplex virus protein VP16 and a synthetic promoter constructed by adding the *tetO* sequence to a minimal promoter [23]. This system was further optimized for application in *A. niger* [24–26] and was also successfully applied in *Fusarium fujikuroi* [27].

For *Trichoderma reesei* two inducible expression systems responding to light [28] or copper [29] were developed. Notably, the inducers also affect the basic biology of *T. reesei* and do not allow the exclusive induction of the target gene(s). In another report, the application of a synthetic expression system that is suitable for a broad range of fungal species including *T. reesei* was described [30]. This system consists of a synthetic transcription factor (TF) and a set of differently strong promoters allowing the expression of gene(s) of interest at different constitutive levels. The synthetic TF itself is a fusion of the DNA-binding protein Bm3R1 from *Bacillus megaterium*, the SV40 nuclear localization signal, and the TAD of VP16 [30]. Please refer to the review by Kluge et al. [31] for a more detailed and comprehensive summary of inducible expression system in filamentous fungi.

In *T. reesei*, the Gal4-like TF Xyr1 (Xylanase regulator 1) is the main activator for the expression of most cellulases and hemicellulases [32]. The main cellulases are the two cellobiohydrolases CBHI and CBHII (EC 3.2.1.91) and the endo-glucanase EGLI (EC 3.2.1.4) [33]. The major hemicellulases are the two endo-ß-1,4-xylanases XYNI and XYNII (EC 3.2.1.8) [34]. Xyr1 is additionally essential for the expression of the aldose reductase Xyl1 (EC 1.1.1.307), which catalyzes the first reaction in the catabolism of certain monosaccharides, such as xylose, galactose, and arabinose [35, 36].

Despite being regulated by the same main transactivator, the expression of cellulases and xylanases is induced under different conditions. Simplified, the expression of the cellulases is induced on lactose, cellulose, and sophorose (transglycosylation product of cellobiose), whereas the expression of the xylanases is induced on xylan and low concentrations of xylose, and partially on lactose [37].

The expression of Xyr1 itself is down-regulated under carbon catabolite repression (CCR), mediated by Cre1 [38, 39] and is induced on cellulase-inducing conditions [40–42]. Recent publications suggest the transcription factors Ace3 and Rxe1 to be involved in the induction of Xyr1 expression [43–45]. Notably, the expression of the Xyr1 target genes is also regulated by further factors and mechanisms, such as the TFs Ace1 [46], Ace2 [47], Ace3 [43], Xpp1 [48] and Rce1 [49], the mating type locus protein Mat1-2-1 [50], the photoreceptor Env1 [51], the protein methyltransferase Lae1 [52], the velvet complex protein Vel1 [53], the Hap2/3/5 complex [54–56], and the MAP kinases Tmk2 [57] and Tmk3 [58].

In a recent study, we constructed a fusion transcription factor (TF), termed XY1, which consists of the N-terminus of Xyr1 and the C-terminus of Ypr1 (Yellow pigment regulator 1) [59], the main activator of sorbicillinoid biosynthesis [60]. Bearing the DBD of Xyr1, the fusion TF XY1 is able to induce the expression of the Xyr1 target genes [59]. The C-terminus of Ypr1 contains the so-called fungal transcription factor middle homology region (FTFMHR), which is responsible for the transactivation [59]. Overexpression of the fusion TF induced the expression of cellulases and xylanases in the Xyr1-deficient recipient strain Xyr1′(81) on different carbon sources, even on d-glucose [59]. The transcript levels of the main xylanase genes *xyn1* and *xyn2*, and the aldose reductase gene *xyl1* reached remarkable high levels and were completely deregulated regarding the carbon source used [59].

In this study we describe the construction of a synthetic TF with the aim to enable controllable expression of the Xyr1 regulon in *T. reesei*. We fused the DBD of Xyr1, the TAD of Ypr1, and the LBD of hERα to create the TF SynX. A constitutive expression cassette for the SynX was inserted into a Xyr1-deficient strain. The tightness of the expression system and the ability of the SynX to induce expression of the main Xyr1 target genes were determined on transcript and enzymatic levels; further we studied the influence of different carbon sources and different concentrations of estradiol on the xylanolytic and cellulolytic activities.

## Results

### 17β-estradiol is suitable for application in *T. reesei*

**Fig. 1:**
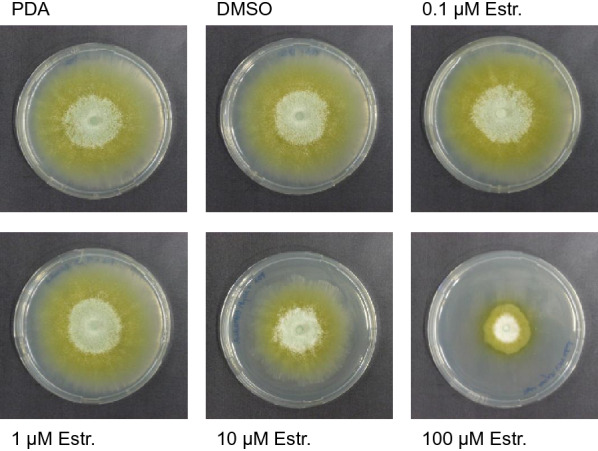
Influence of 17β-estradiol on the growth of *T. reesei*. The wild-type like strain Δ*tmus53*, was cultivated on potato dextrose agar (PDA) plates at 30 °C in darkness for 3 days. 17β-estradiol (Estr.) was added in the given concentrations, and as control only the solvent DMSO

In order to use the hERα in a synthetic transcription factor, the inducing substance 17β-estradiol must not influence the basic biology of the host organism at working concentrations. Consequently, we performed a growth experiment in order to estimate to which extend *T. reesei* can be exposed to 17β-estradiol. The wild-type like strain Δ*tmus53* was cultivated on potato dextrose agar plates in the presence of different concentrations of 17β-estradiol. As a control only the solvent dimethyl sulfoxide (DMSO) was used. After 72 h of growth we observed a growth reduction only in the presence of 10 and 100 µM 17β-estradiol (Fig. 1). For comparison, the 17β-estradiol concentration in the female human body ranges from 20 to 500 pg/mL [61], which corresponds to 0.0734 to 1.835 nM. The hERα in *Aspergillus* sp. responded to similar concentrations [1] as in the human body. In a previously developed synthetic expression system using the hERα in plants, the standard working concentration of 17β-estradiol was 2 µM, however, the system responded to concentrations as low as 8 nM [62]. We concluded that we could use 17β-estradiol in *T. reesei* for a synthetic expression system as long as the working concentration stays within the nM or low µM range.

### Construction of the strain TXYE expressing the synthetic TF SynX

**Fig. 2:**
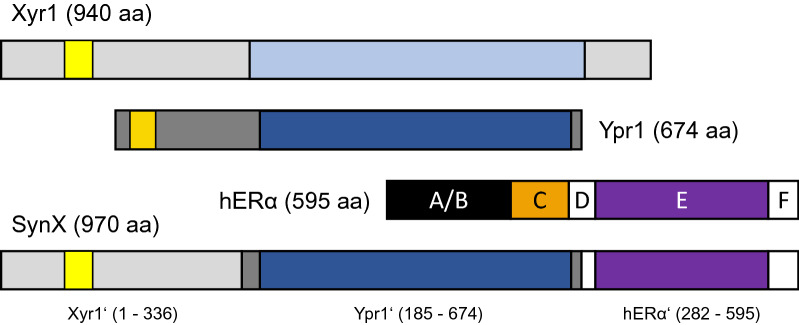
Composition of the synthetic transcription factor SynX. The synthetic transcription factor SynX was constructed by fusing the N-terminal part of Xyr1, containing the Zn(II)2Cys6 binuclear cluster DNA-binding domain (yellow box), the C-terminal part of Ypr1, containing the transactivating fungal transcription factor middle homology region (blue box), and the ligand binding domain of the hERα (domain E, purple box)

**Fig. 3:**
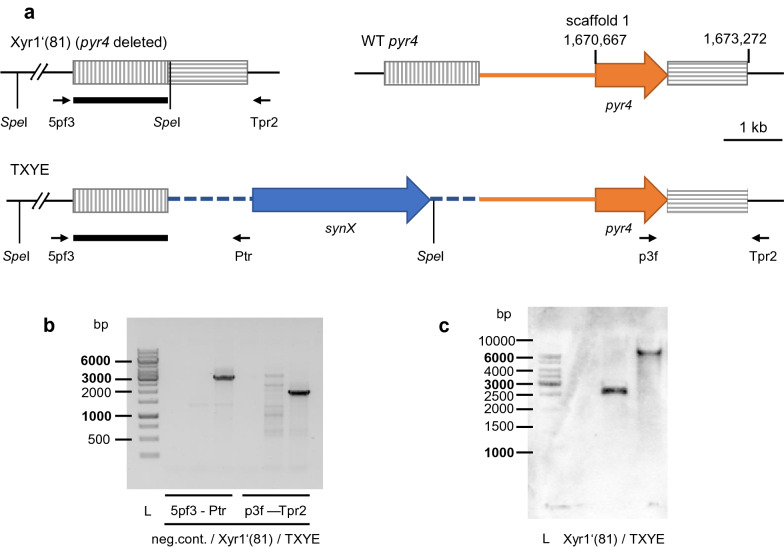
Genomic insertion of the SynX expression cassette. **a** The uridine auxotrophic strain Xyr1′(81) was transformed with the plasmid pRP4-SynX resulting in the targeted integration of the SynX expression cassette (blue arrow and blue, dashed lines) into the *pyr4* locus using the *pyr4* gene (orange arrow) and its promoter (orange line) as auxotrophic marker. The grey boxes represent the flanking regions used for the homologous recombination. The wild-type (WT) *pyr4* locus is depicted for comparison and the chromosomal coordinates according to [74] are given. Position and orientation of the primers used for genotype testing are indicated by short, black arrows. 5pf3, 5pyr4_fwd3; Tpr2, Tpyr4_rev2; Ptr, Ptef_rev-BspTI; p3f, pyr4_3fwd. The thick, black line indicates the hybridization region for the probe used in the Southern blot assay. Recognition sites for the restriction endonuclease *Spe*I are depicted. **b** Agarose gel electrophoresis of the amplification products from PCR assays using the indicated primer pairs and DNA samples of indicated strains. **c** A Southern blot analysis using *Spe*I-digested chromosomal DNA of the indicated strains returned the expected signals at 2501 bp for Xyr1′(81) and 7324 bp for TXYE and verifies the exclusive integration of the expression cassette at the *pyr4* locus. L, DNA size ladder

Xyr1′(81) is a Xyr1-deficient strain that is a useful recipient strain for the overexpression of Xyr1 or of fusion TFs containing the Xyr1 DBD. Xyr1′(81) bears a non-sense mutation in Xyr1 at position 81 and a *pyr4* deletion, which results in an complete abolishment of xylanolytic and cellulolytic activities [59]. In this study, we inserted the synthetic transcription factor SynX into Xyr1′(81). SynX consists of the DBD of Xyr1 (aa 1–336), the TAD of Ypr1 (aa 185–674), and the C-terminal part of hERα (aa 282–595) which contains a nuclear localization signal and the LBD (Fig. 2). The mentioned domains of Xyr1 and Ypr1 were previously successfully combined to form the fusion TF XY1 [59]. The indicated part of the hERα was previously successfully used in a fusion TF in plants [62]. For the construction of SynX, the coding sequence for the hERα LBD was codon-optimized for *T. reesei* (Additional file 1). An overexpression cassette for SynX using the strong, constitutive *tef1* promoter and the terminator of *cbh2* was inserted into the *pyr4* locus by transforming the linearized plasmid pRP4-SynX into strain Xyr1′(81) yielding strain TXYE (Fig. 3a). The correct and single copy integration of the SynX overexpression cassette into the *pyr4* locus was tested by PCR and Southern blot analysis (Fig. 3b, c).

### The synthetic TF SynX induces xylanase expression by addition of 17β-estradiol

**Fig. 4:**
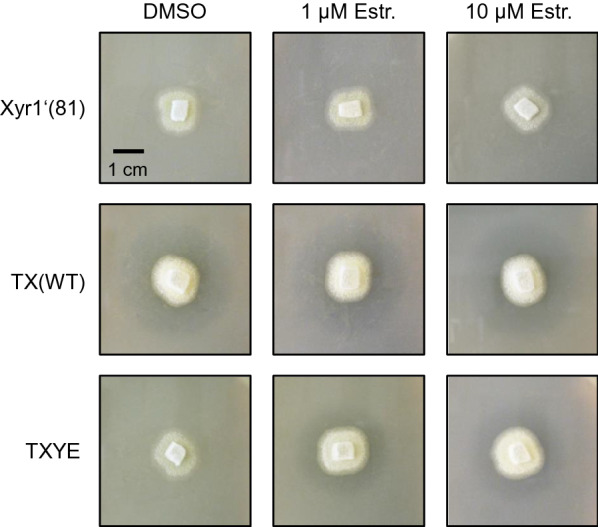
Xylanolytic activities in absence and presence of 17β-estradiol. The Xyr1-deficient strain Xyr1′(81), the Xyr1 overexpression strain TX(WT), and the SynX-bearing strain TXYE were cultivated on xylan plates containing indicated concentrations of 17β-estradiol (Estr.) or only the solvent DMSO as control. Pictures were taken after 72 h of cultivation. Pictures are to scale and can be compared to the scale bar

To test whether SynX can complement for Xyr1-deficiency and induce gene expression of xylanases, we cultivated TXYE, its Xyr1-deficient parent strain Xyr1′(81), and the Xyr1 overexpression strain TX(WT) as positive control on xylan plates. Plates were supplemented with different concentrations of 17β-estradiol dissolved in DMSO and the solvent alone as control (Fig. 4). After 3 days of cultivation we observed no clearing zone around Xyr1′(81), which indicates the absence of xylanolytic activity, confirming previous results [59]. The Xyr1 overexpression strain TX(WT) produced high levels of xylanolytic activity regardless of the 17β-estradiol concentration. A clearing zone around TXYE was exclusively observed in the presence of 17β-estradiol (Fig. 4).

### Determination of the optimal working concentration of 17β-estradiol for SynX activation

**Fig. 5:**
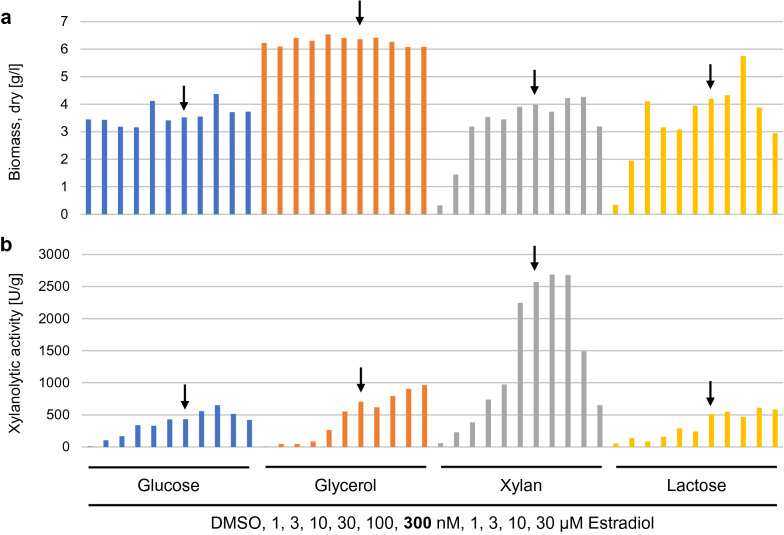
Influence of the concentration of 17β-estradiol on growth and xylanolytic activity. The SynX-bearing strain TXYE was cultivated in shake flasks on the indicated carbon sources containing different concentrations of 17β-estradiol (Estr.) or only the solvent DMSO as control for 48 h. The acquired biomass (**a**) and the endo-xylanolytic activity (**b**) were measured. The arrows indicate the measured values using 300 nM 17β-estradiol

Next, we determined the minimal and optimal working concentration of 17β-estradiol and investigated the possibility of the fine-tuning potential of this synthetic expression system. To this end, we cultivated the SynX expressing strain TXYE in liquid media containing different carbon sources and different concentrations of 17β-estradiol. The carbon sources were chosen according to their impact on the native xylanase expression. Glucose is a repressor of xylanase expression, glycerol is considered to be neutral regarding the expression of xylanases, xylan is a strong natural inducer of xylanase expression, and lactose is a strong inducer of cellulase expression, that can also induce expression of XYNII [55]. We measured the resulting biomass and xylanolytic activity after 48 h of cultivation in shake flasks (Fig. 5). Notably, no growth was observed on xylan and lactose in the absence of 17β-estradiol (Fig. 5a). This can be explained by the 17β-estradiol-dependency of SynX. In wild-type strains, Xyr1 is essential for the expression of the aldose reductase encoding gene *xyl1*, which is in turn essential for growth on xylan and lactose [32, 36]. In TXYE, the 17β-estradiol-sensing SynX replaces the function of Xyr1. On both carbon sources, growth could be restored by addition of only 3 nM 17β-estradiol (Fig. 5a). Interestingly, no growth inhibition was observed at high 17β-estradiol concentrations in the shake flask cultivation (Fig. 5a), in contrast to the observed growth inhibition on plates (Fig. 1).

Regardless of the carbon source used, no xylanolytic activity could be measured in the absence of 17β-estradiol (Fig. 5b), which is in strict accordance with results from the previously described xylan plate assay (Fig. 4). Low xylanolytic activity was already detected at 1 nM 17β-estradiol on all carbon sources tested (Fig. 5b). Further, we observed a 17β-estradiol concentration dependency of the resulting xylanolytic activities. On glucose, the maximum xylanolytic activity was approx. 650 U/g at 3 µM 17β-estradiol, on glycerol approx. 950 U/g at 30 µM, on xylan approx. 2700 U/g at 1 µM, and on lactose approx. 615 U/g at 10 µM 17β-estradiol. On glucose and xylan, high 17β-estradiol concentrations (10 µM and 30 µM) had a negative impact on the resulting xylanolytic activities (Fig. 5b). We decided to use 300 nM as standard working concentration because on xylan and lactose the measured xylanolytic activities were very close to the respective maximum activity (Fig. 5b), while on glucose and glycerol the maximum activities were reached at 17β-estradiol concentrations that we considered to be too high for a feasible application.

### SynX induces expression of xylanases at high levels but hardly any cellulases

**Fig. 6:**
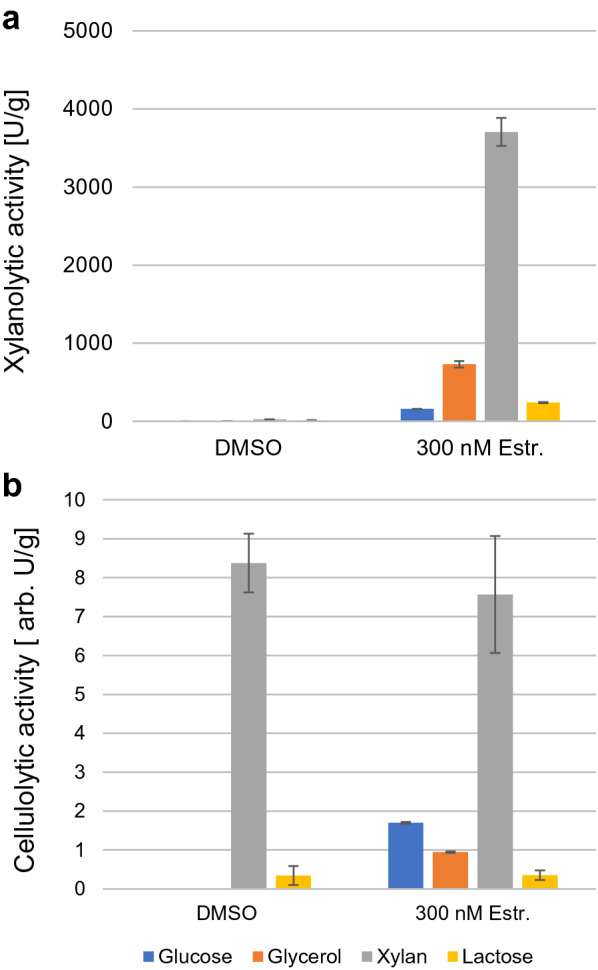
Xylanolytic and cellulolytic activity of TXYE using 300 nM 17β-estradiol. The SynX-bearing strain TXYE was cultivated in shake flasks on the indicated carbon sources containing 300 nM 17β-estradiol (Estr.) or only the solvent DMSO as control. Samples were taken after 72 h. The endo-xylanolytic activity (**a**) and the endo-cellulolytic activity (**b**) were measured in the supernatants and normalized to the acquired biomass. The values provided in the figures are means from three biological replicates. Error bars indicate standard deviations

We performed a similar experiment with the optimal 17β-estradiol concentration of 300 nM in biological triplicates and for a longer cultivation period (72 h) to validate the results from the experiment before (48 h cultivation time, no replicates). Again, we measured no xylanolytic activities in the absence of 17β-estradiol, confirming the tightness of the system (Fig. 6a). In the presence of 300 nM 17β-estradiol, the xylanolytic activities in the supernatant activities reached 160, 730, 3706, and 239 U/g on glucose, glycerol, xylan, and lactose, respectively (Fig. 6a). As mentioned previously, Xyr1 is also the main activator for the expression of cellulases in *T. reesei*. Consequently, we were interested in the influence of SynX on the expression of cellulases. We measured the total cellulolytic activity using resorufin-beta-d-cellobioside as substrate and detected only low cellulolytic activity (Fig. 6b). On xylan, approx. 8 arbitrary U/g of cellulolytic activity were detected regardless if 17β-estradiol was added or not (Fig. 6b). This is in the same range as the activity induced by XY1 in our previous study (approx. 10 arbitrary U/g, see [59]). On lactose, only very low activity was detected, regardless if 17β-estradiol was added or not (Fig. 6b). The fusion TF XY1 triggered production of over 120 arbitrary U/g of cellulolytic activity in the same experimental setup [59]. On glucose and glycerol, the expression of cellulases was slightly induced by SynX in the presence of 300 nM 17β-estradiol resulting in approx. 1.7 and 1 arbitrary U/g, respectively (Fig. 6b). For comparison, the fusion TF XY1 resulted in approx. 10 and 40 arbitrary U/g in the same experimental setup [59].

### SynX upregulates transcription of genes encoding for the main xylanase and the aldose reductase Xyl1

Next, we wanted to get a more detailed insight into the transactivation activity of SynX and its abilities to induce gene expression of the Xyr1 regulon. To this end, strain TXYE was pre-grown on Mandels-Andreotti (MA) medium [63] containing glycerol as the carbon source without estradiol, because this condition represents a non-induced, non-repressed state. After 24 h of pre-cultivation, equal amounts of mycelium were transferred to MA media containing glucose, glycerol, xylose (mimics induction on xylan [64]), or lactose, and MA medium without carbon source as a control. The MA media were supplemented with 300 nM 17β-estradiol or only with the solvent DMSO as a control. Samples were taken after 3 and 6 h of cultivation and the total RNA was extracted. A reverse transcription quantitative PCR (RT-qPCR) assay was performed to measure relative transcript levels of the main Xyr1 target genes.

**Fig. 7:**
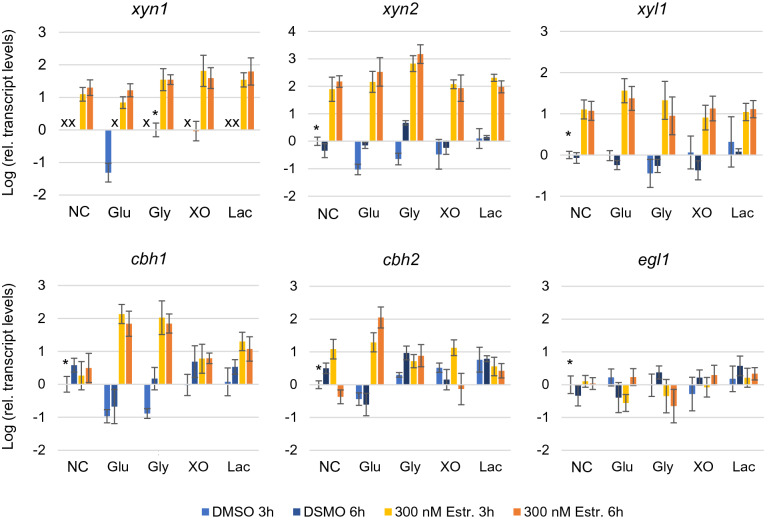
Transcript levels of the main cellulases-, xylanases- and the aldose reductase encoding genes. The SynX-bearing strain TXYE was pre-cultivated on in shake flasks on glycerol without 17β-estradiol for 24 h. Acquired mycelium was filtered and washed and then equal amounts were transferred to medium containing no carbon source (NC), glucose (Glu), glycerol (Gly), 1 mM xylose (XO), or lactose (Lac) and either 300 nM 17β-estradiol (Estr.) or only the solvent DMSO as control. Samples were taken after 3 and 6 h of incubation, the total RNA was isolated and the relative transcript levels of the indicated genes were determined by a RT-qPCR assay normalized to the reference sample (indicated by an asterisk) using the reference genes *sar1* and *act1* for normalization. X represents no detection. Error bars indicate standard deviation from three independently grown cultures

SynX was able to induce the gene expression of the two main xylanases (*xyn1* and *xyn2*) and the aldose reductase *xyl1* in dependency of 17β-estradiol on all tested carbon sources (Fig. 7). For all three tested genes no or only very low transcript levels were detected when supplemented with DMSO only (Fig. 7).

The induction of gene expression of the main cellulases, *cbh1*, *cbh2* and *egl1* by SynX painted a different picture. We measured high transcript levels of *cbh1* on glucose and glycerol in the presence of 17β-estradiol after 3 and 6 h (Fig. 7). On lactose moderately elevated *cbh1* transcript levels were measured in the presence of 17β-estradiol. Without carbon source and on xylose, no induction could be achieved by the addition of 17β-estradiol (Fig. 7). The transcript levels of *cbh2* were distinctively elevated on glucose in the presence of 17β-estradiol. The other measured *cbh2* transcript levels are within a single log-unit (Fig. 7). The same is true for all measured transcript levels of *egl1*. Notably, the obtained Ct values were all close to the limit of detection. SynX did not induce gene expression of *egl1*, regardless of carbon source or the presence of 17β-estradiol (Fig. 7). These results match the previously measured enzymatic activities to the greatest part, i.e. substantial xylanolytic and only low cellulolytic activities (Fig. 6).

## Discussion

Previously, the complete hERα was used in filamentous fungi (*Aspergillus* spp.) [1], and the LBD of hERα was used in plants in combination with the TAD of VP16 [62]. In this study, we used a synthetic biology approach to evaluate the potential of the LBD of hERα to be used in combination with a TAD from an endogenous TF (i.e. Ypr1) for inducible gene expression in filamentous fungi. We demonstrated earlier that the TAD of Ypr1 can be used to induce the gene expression of Xyr1 target genes nearly carbon source independently by fusing it to the DBD of Xyr1, resulting in the fusion TF XY1 [59]. Here, we further added the LBD of the hERα, resulting in the synthetic TF SynX, with the aim to gain control over the induction process.

Using enzyme activity and RT-qPCR assays, we demonstrated that SynX induces the gene expression of xylanases and the aldose reductase Xyl1 encoding genes in dependency of its inducer 17β-estradiol on any tested carbon source. The induction regulatory system seems to be tight, as we could not detect transcripts or enzyme activities in the absence of 17β-estradiol.

On the other hand, SynX was not able to induce the expression of cellulases to extends worth mentioning. Previously, an overexpression of Xyr1 resulted in a complete abolishment of cellulase production on lactose and elevated cellulolytic activity formation on xylan, whereas the overexpression of the fusion TF XY1 led to elevated cellulolytic activities on lactose and lower levels on xylan than in the Xyr1-overexpression strain [59]. Compared to the enzyme activities in that previous study, we detected moderate cellulolytic activity on xylan and only very low cellulolytic activity on lactose regardless of the presence of 17β-estradiol in TXYE (Fig. 6b) in this study. On glucose and glycerol, the addition of 17β-estradiol resulted in relatively low cellulolytic activities compared to xylan (Fig. 6b). Thus, the 17β-estradiol-independent cellulolytic activities on xylan and lactose could simply be the result of release from CCR maybe in combination with chromatin opening and the action of other transcription factors. This again demonstrates that the regulation of cellulase expression is a highly complex topic, which still offers surprises and future research possibilities.

The measured *cbh1*, *cbh2*, and *egl1* transcript levels only partly matched the low cellulolytic activities. We detected virtually no *egl1* transcripts on all carbon sources, regardless of the usage of estradiol. Induction of the exo-cellulase encoding genes *cbh1* and *cbh2* was observed on glucose and glycerol upon addition of estradiol (Fig. 7). Notably, this induction was a lot less pronounced compared to the one obtained by using TF XY1 in our previous study [59]. The replacement on xylose did not induce genes expression of the cellulase encoding genes compared to a replacement to no carbon source (Fig. 7), but we could measure enzyme activity on xylan (Fig. 6b). We reason that the induction experiment with xylose does not simulate the cultivation on xylan entirely.

When we compare the promoter architecture of xylanases and cellulases, we can observe a striking difference. In the xylanase promoters, the Xyr1-binding sites are positioned as inverted repeats, as expected for Gal4-like TFs [65]. In the cellulase promoters on the other hand, the Xyr1-binding sites are arranged as direct repeats [65]. Upon the insertion of an inverted repeat of Xyr1-binding sites into the *cbh1* promoter, the CBHI expression was induced on xylan [65]. These results might indicate that Xyr1 has two different modes of action. We suggest the following hypothesis: Xyr1 forms homodimers to bind to the inverted repeats in the xylanase promoters and binds the tandem repeats in the cellulase promoters in a different way. Notably, the native hERα is binding to palindromic sequences (can be viewed as inverted repeats) upon the formation of homodimers [6, 66]. The presence of the LBD of hERα in SynX enforces the formation of homodimers that preferably induce the expression of xylanases but not cellulases.

The measured xylanolytic activities were on average lower in the SynX-bearing strain TXYE than in the before mentioned XY1-bearing strain TXY(1) [59]. For example, TXY(1) reached xylanolytic activities of over 10,000 U/g on glycerol [59], whereas TXYE yielded only approx. 950 U/g in the presence of 300 nM 17β-estradiol (Fig. 6a). Notably, the used 17β-estradiol concentration (300 nM) was not the optimal concentration for induction of the xylanases on glycerol (Fig. 5b). On xylan on the other hand, TXYE secreted higher amounts of xylanases (approx. 3700 U/g, Fig. 6a) than TXY(1) (approx. 2500 U/g [59]).

These results may indicate a generally lower transactivation capability of SynX than XY1. A simple explanation for this might be unwanted changes of the secondary and/or tertiary structure by intra-molecular interactions of the three domains in SynX. Alternatively, it can be assumed that the LBD of hERα might interfere with protein–protein-interactions between the TAD of Ypr1 and the transcription machinery and/or additional activating factors. Further, we have to consider the influence of other regulatory factors and mechanisms that are responding exclusively to the carbon source, but not to 17β-estradiol. Notably, not only Xyr1 but also other regulatory factors and mechanism play important roles in the regulation of expression of xylanases and cellulase encoding genes, i.e. the transcription factors Cre1 [38, 39, 67], Ace1 [46], Ace2 [47], Ace3 [43], Xpp1 [48] and Rce1 [49], the mating type locus protein Mat1-2-1 [50], the photoreceptor Env1 [51], the protein methyltransferase Lae1 [52], the velvet complex protein Vel1 [53], the Hap2/3/5 complex [54–56], and the MAP kinases Tmk2 [57] and Tmk3 [58]. Further, we observed in a previous study that the DNA packaging adds an important layer to the regulation of expression of xylanase encoding genes [68]. It seems as if *T. reesei* is integrating a lot of different stimuli using this many different regulators. At the moment, we can only speculate which regulatory factors and/or mechanisms are mediating the carbon source signal (xylan vs. glycerol) and interfere with the induction of cellulase expression by SynX.

## Conclusions

In this study we constructed and expressed the synthetic TF SynX (consisting of the DBD of Xyr1, the TAD of Ypr1 and the LBD of the hERα) in *T. reesei*. SynX successfully induced gene expression of xylanases upon induction with 17β-estradiol and did not activate gene expression without 17β-estradiol. This demonstrates that the LBD of the hERα can be used as inducible regulatory domain in *T. reesei*. Further, we could demonstrate that the LBD of the hERα is also functional when fused to a TAD other than the commonly used TAD of VP16.

## Methods

### Fungal strains and cultivation conditions

All *T. reesei* strains (Table 1) used in this study were maintained on malt extract agar at 30 °C. Uridine and Hygromycin B were added when applicable to a final concentration of 5 mM and 113 U/mL, respectively. 17β-estradiol (Sigma-Aldrich, part of Merck KGaA, Darmstadt, Germany) was added in the given concentrations. For each concentration, a separate 1000-fold stock solution in dimethylsulfoxid (DMSO) was prepared in order to be able to add equal volumes of the 17β-estradiol solution and thereby excluding the potential influence of the amount of added solvent.

**Table 1:** *T. reesei* strains used in this study

Designation	Description	Source
QM6a Δ*tmus53*	wild-type-like strain with deficiency of the non-homologous end joining repair pathway	[73]
Xyr1′(81)	Xyr1-deficient strain due to a non-sense mutation at position 81 of Xyr1; *pyr4* deleted background, no xylanolytic nor cellulolytic activity	[59]
TX(WT)	Overexpression of Xyr1; *xyr1* under the control of the *tef1* promoter inserted at the *pyr4* locus of Xyr1′(81); uridine prototrophy re-established	[59]
TXYE	Expression of the fusion TF SynX; the fusion gene under the control of the *tef1* promoter inserted at the *pyr4* locus of Xyr1′(81); uridine prototrophy re-established	this study

For cultivations on potato dextrose agar (PDA) plates, *T. reesei* was pre-grown on PDA plates at 30 °C for 3 days in darkness. Then equally sized (approx. 0.3 cm diameter) overgrown pieces of agar were transferred to fresh PDA plates containing different concentrations of 17β-estradiol and incubated at 30 °C in darkness for further 3 days.

For cultivations on xylan plates, *T. reesei* was pre-grown on Mandels-Andreotti (MA) medium (8.9 g/L Na_2_HPO_4_∙2 H_2_O, 1.4 g/L (NH_4_)_2_SO_4_, 2 g/L KH_2_PO_4_, 0.3 g/L MgSO_4_, 0.4 g/L CaCl_2_, 0.3 g/L urea, 1 g/L peptone, 20 mL/L trace elements (5 mg/L FeSO_4_∙7 H_2_O, 1.6 mg/L MnSO_4_∙H_2_O, 1.4 mg/L ZnSO_4_∙H_2_O and 2 mg/L CoCl_2_∙2 H_2_O), pH adjusted to 5 with citric acid) [63] plates containing 1% (w/v) xylan from beechwood (Carl Roth GmbH + Co KG, Karlsruhe, Germany) at 30 °C for 3 days in darkness. Then equally sized (approx. 0.3 cm diameter) overgrown pieces of agar were transferred to fresh plates containing additionally 0.1% (v/v) Igepal (Carl Roth GmbH + Co KG) and the plates were incubated at 30 °C in darkness.

For cultivations in shake flasks, *T. reesei* was grown in 50 ml MA medium containing 1% (w/v) glucose monohydrate, glycerol, xylan from beechwood (Carl Roth), or lactose at 30 °C on a rotary shaker at 180 rpm. A total of 10^9^ conidia per liter (final concentration) was used as the inoculum. Mycelia and supernatants were separated by filtration through Miracloth (EMD Millipore, part of Merck KGaA, Darmstadt, Germany). Mycelia were dried at 80 °C over night for biomass determination and supernatants were stored at − 20 °C.

For the replacement experiment, *T. reesei* was pre-grown in 200 ml MA medium containing 1% glycerol at 30 °C on a rotary shaker at 180 rpm. A total of 10^9^ conidia per liter (final concentration) was used as the inoculum. Pre-grown mycelia were washed with sterile tap-water, and equal amounts (approx. 0.5 cm^3^) were resuspended in 20 ml MA medium containing the indicated additives and cultivated at 30 °C on a rotary shaker at 180 rpm.

### Plasmid constructions

PCRs for cloning purposes were performed with Q5 High-Fidelity DNA Polymerase (New England Biolabs (NEB), Ipswich, MA, USA) according to the manufacturer’s instructions. All used primers are listed in Table 2. PCR products were cloned into *Eco*RV-digested pJET1.2 (Thermo Scientific, part of Thermo Fisher Scientific Inc., Waltham, MA, USA) and after verification of the PCR products by sequencing (Microsynth, Balgach, Switzerland), they were released for subsequent cloning purposes by digestion with suitable restriction endonucleases (NEB).

**Table 2:** Primers used in this study

Name	Sequence (5′-3′)
Ypr1_L185f-VspI	ATTAATCTTACTCCACAGTCGACAACG
Ypr1_G674r-MfeI	CAATTGCGCCGTAAATGCTCCCATCG
5pyr4_fwd3	CCAGACGGTGATTCACATATACG
Ptef_rev-BspTI	CTTAAGTGTGATGTAGCGTGAGAGCTG
pyr4_3fwd	AGACGAGGACCAGCAGACC
Tpyr4_rev2	CAGGAAGCTCAGCGTCGAG
5pyr4_fwd(BglII)	GCGGAAGATCTCGAGATAGTATCTC
5pyr4_rev-BspEI	TCCGGAGTAGCTCTTCACTGGTTGTGGTG
sar1fw	TGGATCGTCAACTGGTTCTACGA
sar1rev	GCATGTGTAGCAACGTGGTCTTT
act1f	TGAGAGCGGTGGTATCCACG
act1r	GGTACCACCAGACATGACAATGTTG
cbh1f	GATGATGACTACGCCAACATGCTG
cbh1r	ACGGCACCGGGTGTGG
cbh2f	CTATGCCGGACAGTTTGTGGTG
cbh2r	GTCAGGCTCAATAACCAGGAGG
egl1f	CTGCAACGAGATGGATATCCTGG
egl1r	GTAGTAGCTTTTGTAGCCGCTGC
xyn1f	CAGCTATTCGCCTTCCAACAC
xyn1r	CAAAGTTGATGGGAGCAGAAG
xyn2_q1f	CCGTCAACTGGTCCAACTCG
xyn2_q1r	GTGCGGTAAATGTCGTAGACG
xyl1-fwd	CTGTGACTATGGCAACGAAAAGGAG
xyl1-rev	CACAGCTTGGACACGATGAAGAG

For the construction of pRP4-SynX, first, the codon-optimized coding sequence of the hERα part (aa 282–595, Additional file 1, gene synthesis was performed by BioCat GmbH, Heidelberg, Germany) was inserted into the plasmid pJET-Ptef-xyr1N [59] via digestion with *Mfe*I and *Nhe*I. Next, the coding sequence for the C-terminal part of Ypr1 was amplified by PCR using the primer Ypr1_L185f-VspI and Ypr1_G674r-MfeI and as template cDNA of *T. reesei* Δ*tmus53* grown on glucose, and then inserted into the latter plasmid via digestion with *Mfe*I and *Nhe*I. The Ptef::*xyr1*::*hER*::*ypr1* fragment was released from the resulting plasmid by digestion with *Kpn*2I and *Spe*I and inserted into the accordingly digested pCD-RPyr4T [69].

### Fungal transformations

The protoplast generation and transformation of *T. reesei* was performed as described previously [70]. Typically, 10 µg of linearized plasmid DNA (in 15 µL sterile ddH_2_O) was used for the transformation of 10^7^ protoplasts (in 200 µL). Selection was performed as described previously [69]. Resulting candidates were subjected to homokaryon purification by streaking conidia on plates with selection medium.

### Isolation of chromosomal DNA

Chromosomal DNA was isolated from mycelium by grinding in liquid nitrogen followed by a phenol/chloroform extraction [70]. RNA was degraded using RNaseA (Thermo Scientific). DNA was precipitated with isopropanol, washed with 70% ethanol, and dissolved in ddH_2_O.

### Genotype testing by PCR

For testing the genotype, 10 ng of chromosomal DNA were used as template in a 25-µL-PCR using OneTaq polymerase (NEB) according to the manufacturer’s instructions. All used primers are listed in Table 2. For the agarose gel electrophoresis of the amplification products the GeneRuler 1 kb DNA Ladder was applied (Thermo Scientific).

### Southern blot analysis

15 µg of chromosomal DNA were digested with 30 U *Spe*I (NEB). The resulting DNA fragments were separated by electrophoresis on an 0.8% agarose gel using the GeneRuler 1 kb DNA Ladder for size estimation, then denatured in 0.4 M NaOH, and transferred by capillary forces onto a Biodyne B 0.45 µm nylon membrane (Pall Corporation, Port Washington, NY, USA) using 10 × SSC. 1.5 µg of biotinylated DNA probe were used for hybridization at 65 °C overnight. The probe was generated by PCR with the primers 5pyr4_fwd(BglII) and 5pyr4_rev-BspEI using chromosomal DNA of *T. reesei* Δ*tmus53* as template. Labeling of the probe was performed by using a Klenow Fragment (exo-) (Thermo Scientific), random hexamer primers, and biotin-11-dUTP (Jena Bioscience, Jena, Germany). Signals were visualized by using Poly-HRP conjugated to streptavidin and ECL Plus Western Blotting substrate (both Thermo Scientific) on a ChemiDoc MP (Bio-Rad Laboratories, Hercules, USA).

### Determination of enzymatic activities

Endo-xylanolytic activities of cultivation supernatants were measured with Azo-Xylan (Megazyme International Ireland, Wicklow, Ireland) according to the manufacturer’s instructions. One unit of activity is defined as the amount of enzyme required to release one μmol of reducing-sugar-equivalents per minute.

Total cellulolytic enzyme activity of cultivation supernatants were measured using the Cellulase Activity Assay kit (Fluorometric) (abcam189817, Abcam PLC, Cambridge, UK) according to the manufacturer’s instructions, with the following adaptions: fluorescence was measured on a Promega GloMax Multi Detection system using the green filter cube (Ex: 520 nm, Em: 580–640 nm), measured fluorescence change rate (Δfluo/min) was used to calculate arbitrary units/ml by multiplying Δfluo/min with 5.1136*10^−4^. Measurements were performed in technical duplicates.

### RNA extraction

0.01–0.03 g of harvested mycelia were homogenized in 1 mL of peqGOLD TriFast DNA/RNA/protein purification system reagent (VWR, part of Avantor Performance Materials, LLC, Radnor, PA, USA) using a FastPrep FP120 BIO101 ThermoSavant cell disrupter (Qbiogene, Carlsbad, US). RNA was isolated according to the manufacturer’s instructions, and the concentration was measured using the NanoDrop ONE (Thermo Scientific).

### Transcript analysis by RT-qPCR

1 µg of isolated RNA were subjected to a DNaseI treatment (Thermo Scientific) according to the manufacturer’s instructions and then reverse transcribed using the LunaScript RT SuperMix (NEB) also according to the manufacturer’s instructions. The cDNA was diluted 1:50 and 2 µL were used as template in a 15 µL reaction using the Luna Universal qPCR Master Mix (NEB) according to the manufacturer’s instructions. All reactions were performed in triplicates on a Rotor-Gene Q system (Qiagen, Hilden, Germany). Calculations of the relative transcript levels were performed according to the Double Delta Ct method [71] using the reference genes *sar1* and *act1* for normalization according to [72].

## **Supplementary information**

**Additional file 1.** Partial coding region of the hERα (aa 282–595), codon-optimized for *T. reesei*, including recognition sites for restriction endonucleases for cloning purposes.

## Data Availability

All data and materials described are freely available for scientific and academic purposes upon request to the corresponding author.

## Abbreviations

CCR: Carbon catabolite repression
DBD: DNA-binding domain
DMSO: Dimethyl sulfoxide
FTFMHR: Fungal transcription factor middle homology region
hERα: Human estrogen receptor α
LBD: Ligand binding domain
PDA: Potato dextrose agar
TAD: Transactivation domain
TF: Transcription factor
RT-qPCR: Reverse transcription quantitative PCR
